# SeMSA: a compact super absorber optimised for broadband, low-frequency noise attenuation

**DOI:** 10.1038/s41598-020-73933-0

**Published:** 2020-10-21

**Authors:** Andrew McKay, Ian Davis, Jack Killeen, Gareth J. Bennett

**Affiliations:** 1 Department of Mechanical and Manufacturing Engineering, Trinity College Dublin, The University of Dublin, Dublin, Ireland; 2 Efficient Energy Transfer (ηET) Department, Nokia Bell Labs, Blanchardstown Business and Technology Park, Dublin 15, Ireland

**Keywords:** Engineering, Materials science, Physics

## Abstract

The attenuation of low-frequency broadband noise in a light, small form-factor is an intractable challenge. In this paper, a new technology is presented which employs the highly efficient visco-thermal loss mechanism of a micro-perforated plate (MPP) and successfully lowers its frequency response by combining it with decorated membrane resonators (DMR). Absorption comes from the membranes but primarily from the MPP, as the motion of the two membranes causes a pressure differential across the MPP creating airflow through the perforations. This combination of DMR and MPP has led to the Segmented Membrane Sound Absorber (SeMSA) design, which is extremely effective at low-frequency broadband sound absorption and which can achieve this at deep sub-wavelength thicknesses. The technology is compared to other absorbers to be found in the literature and the SeMSA outperforms them all in either the 20–1000 Hz or 20–1200 Hz range for depths of up to 120 mm. This was verified through analytical, finite element and experimental analyses.

## Introduction

The attenuation of low frequency, broadband noise remains an intractable challenge both in the domains of fundamental and applied physics where the attenuation of acoustic waves can require the treatment of wavelengths of many meters in length. Regarding sound absorption, porous materials such as open-cell polyurethane and melamine foams effectively attenuate broadband noise. However, their efficiency is a function of their overall thickness, thus rendering them impractical at low frequencies for many real applications. Regarding sound isolation, barriers and panels whose efficiency increases with their mass similarly become infeasible at low frequencies due to excessive weight.

An alternative approach to porous materials and stiff panels employs resonance which can be highly efficient but tends to be tonal in response. However, the combination of a microperforated panel (MPP) with a backing cavity has resulted in one of the most successful broadband sound-absorbing technologies to date and has been examined thoroughly by researchers such as Maa^1^. The MPP on its own consists of a thin panel perforated by sub-millimetre holes which is then backed by a sealed cavity. The combination behaves as a Helmholtz resonator where the mass is the mass of air in the hole and the spring is the compliance of the volume of air in the cavity. Acoustic energy is converted to heat in the acoustic boundary layer formed within the small perforations. To maximise these losses the hole radius is chosen to be approximately equal to the viscous penetration depth $\delta =\sqrt{\frac{2\mu _0}{\omega \rho _0}}$ where $\mu _0$ is the viscosity of air, $\omega$ is the angular frequency and $\rho _0$ is the density of air. The MPP produces a reasonably broad absorption peak compared to many other resonant technologies and the peak is readily tailored through changes to the panel thickness, hole diameter, hole spacing and cavity depth. As well as being an effective absorber it is also simple to manufacture and very robust, all of which have made it an extremely popular choice for sound absorption. However, whilst being an excellent broadband absorber, the cavity depth is the parameter which controls its lower frequency limit and thus for this solution also, its size becomes unreasonable when designed to address low-frequency noise.

Acoustic metamaterials (AMMs) provide alternative approaches to sound attenuation compared with those of traditional materials. This is often accomplished by exploiting engineered sub-wavelength components, sometimes called “meta-atoms” or inclusions, which can control sound whose wavelengths are orders of magnitude longer than these AMM sub-structures. The emerging AMMs can therefore not only address the sample size problem but can also exceed known bounds on conventional material properties. However, most of the literature to date present technologies with impressive properties at low frequencies but which typically are only effective at tones or in narrow frequency bands. Dissipation can be enhanced by increasing the energy density within a material and so AMMs capitalise on this by successfully creating resonances of these inclusions within the material itself. There are many examples in the literature where small scale resonant inclusions achieve near-perfect absorption or reflection at low frequencies. This began with one of the first examples of an AMM^2^ and has progressed to the development of other resonant technologies and oscillating membranes^3^, the latter of which is of interest in the current research.

When small masses are attached to these membranes they tend to be called Decorated Membrane Resonators (DMRs). A single negative DMR, with dipolar symmetry, can attain negative mass density for perfect reflection^3^ or near-zero mass density leading to near-perfect impedance matching/super coupling which results in near-perfect transmission^4^. Given the possibilities to fabricate these membranes from sub-millimetre elastomeric films, they have extraordinary potential to be lightweight and thin, high transmission loss (TL) panels, and have been proposed as a light, thin solution in aviation to reduce cabin noise for example^5^. Optimising the configuration of the attached masses has resulted in broader bandwidths for TL^6^ or indeed absorption^7^ of the noise into the membrane itself. Constraining the elastomeric membranes in some way with a rigid support^8^ has resulted in an alternative method to increase the modal density of vibration.

In this paper, a new technology is presented which employs the highly efficient visco-thermal loss mechanism of an MPP and successfully lowers its frequency response by combining it with decorated mass resonators. The combination of the two successfully results in both a low frequency and broadband solution for sound absorption. An equivalent circuit analytical model is developed and compared to both finite element analysis and experimental results.

Approximately two decades after the first AMM was experimentally validated, engagement in this highly active research field has resulted in a significant body of published work. However, what seems to be absent, in contrast to other research areas, is an attempt to compare the performance of technologies as a function of certain parameters such as depth. An additional contribution of this work is a simple benchmarking exercise of the best performing low frequency, broadband sound absorption solutions to be found in the literature to the authors’ best knowledge. The exercise shows that the current technology, through an optimisation routine of the equivalent circuit model, results in the best performing sound-absorbing solution in the 20–1200 Hz range for a wide range of technology depths.

## Segmented membrane sound absorber (SeMSA)

**Figure 1:**
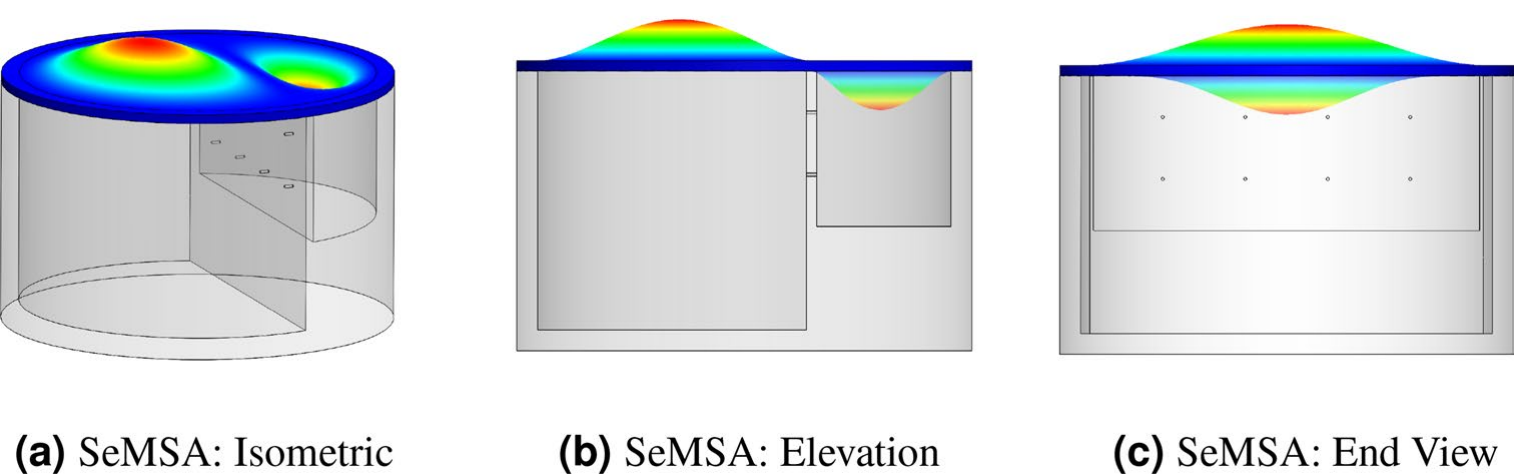
Segmented membrane sound absorber (SeMSA). General concept schematic.

**Figure 2:**
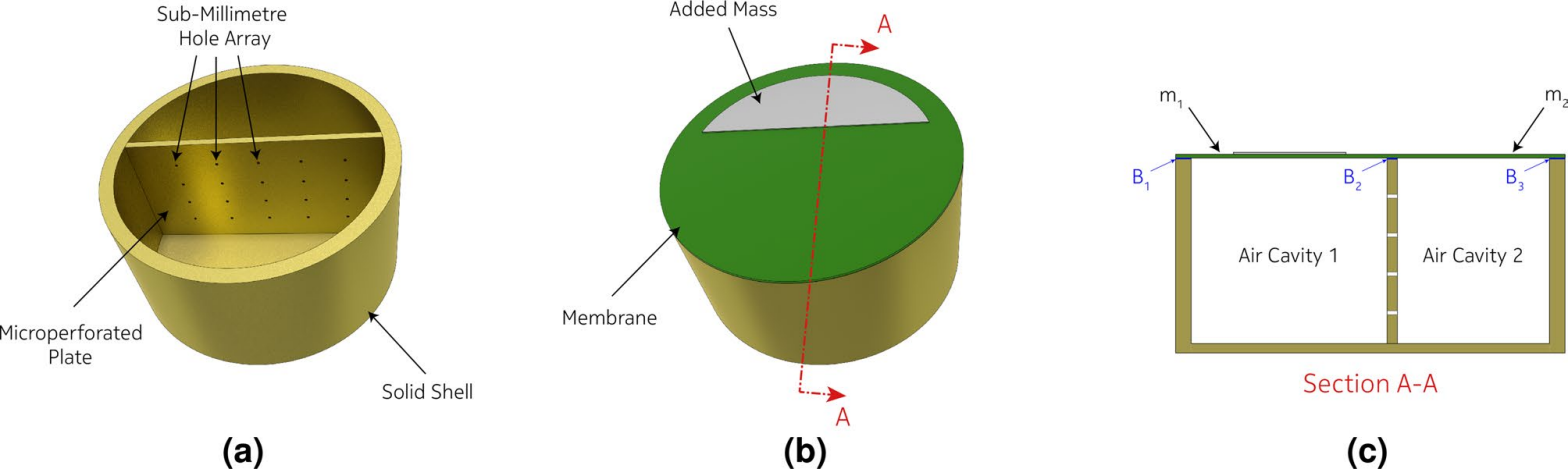
Schematic of a cylindrical SeMSA unit cell.

Figures 1 and 2 present a single cell of the novel SeMSA technology. The cell consists of two chambers of solid walls and bases separated by an MPP. The top surface of the cell is sealed by a sub-millimetre elastomeric membrane. The sealing is performed not only around the perimeter of the cell but also along the top of the dividing MPP plate resulting effectively in two separate membranes. Additional masses, shown in Fig. 2 for example, can be attached to one or both membranes resulting in total mass values, including the masses of the membranes themselves, of $m_1$ and $m_2$. The depths of the chambers can vary relative to each other and the position of the MPP is typically offset from the central axis. Without perforations, each chamber in isolation would operate as an approximate simple mass/spring oscillator at its lower mode. The addition of the masses allows their resonant frequencies to be easily lowered to the frequency range of interest. We know that these oscillators can be implemented through reference to the DMR literature so that they could potentially be either: perfect reflectors in a narrow frequency range; or even absorbers at higher membrane eigenmodes if the masses were sized and located to result in a “flapping” motion of the asymmetric added masses^7^. For the latter, the absorption would result from acoustic energy transfer into the membrane if the membrane was caused to stretch due to the mass movement. In our work, although we consider damping due to motion of the membrane, the dominant absorption mechanism is due to the MPP and to facilitate the development of an analytical model, which is absent from the work of Mei et al.^7^, we examine only the “limp mass” behaviour of the membranes under no tension and whose dynamic response is dominated by their inertial response with negligible membrane stiffness effects. Practically, this means that the added mass must be sized slightly smaller than the cell segment so that there is a latex “hinge” around the mass to ensure limp mass behaviour where, for the first mode, the mass oscillates parallel to the membrane with no rotation. The membrane is not pre-tensioned and the dynamic displacement is sufficiently low to minimise stretching in the membrane at the hinge. The significant benefit of this approach with the development of an analytical model is that it lends itself to a less complex optimisation routine than would be possible when considering membrane effects. This routine is then used to determine the optimal SeMSA parameters for maximum absorption.

The basic operating principle of the SeMSA absorber is that an acoustic wave that is normally-incident upon the membranes will excite piston-like motions of the membranes. The resonance frequencies of each membrane and air cavity system will be dictated by the membrane masses and stiffnesses of the air inside the cavities. However, as the two membrane-cavity systems are separated by the MPP, in the lower frequency range, the total coupled system is a 2 degree of freedom system whose resonances differ to those of the isolated chambers and where significant damping is now added due to the perforations in the MPP. The absorption profile can be tailored by adjusting parameters such as the membrane mass in each segment, parameters related to the MPP or the relative volume of the segments. The MPP separating the segments allows control over the damping in the system as the out-of-phase motion of the two membranes causes a pressure differential across the MPP creating airflow through the perforations. It should be noted that as the two cavities usually differ in volume/stiffness that in-phase motion can also result in a pressure differential across the MPP.

## Benchmarking low frequency, broadband sound absorbers

The performance of a sound absorber is typically characterised by the absorption coefficient as a function of the depth of the absorber in relation to the acoustic wavelength, $\lambda$. For absorptive foams such as polyurethane or melamine foams, depths of between $\lambda$ and $\lambda /4$ are required to achieve significant absorption. As mentioned, acoustic metamaterials have provided a step-change in technological development by providing sound absorption with depths in the order of $<\lambda /10$^9–11^ in some cases. When noise reduction technologies are actually implemented for noise abatement, a sound absorber is used to cover a large surface or at least one whose area is many times the area of a unit cell of a metamaterial, and so a volumetric consideration reduces to a depth constraint. Our suggested metric for comparison between candidate technologies is then the linear average absorption in the chosen frequency range per unit depth. For reasons of brevity we will simply use the term average absorption to mean linear average absorption from here on in. This metric is closely related to the causality constraint of Yang et al.^12^ which gives the minimum depth of material that is required to achieve a desired absorption performance.

In this section, an industry-standard acoustic foam and some of the best performing low-frequency broadband AMMs as published in the literature are compared against each other as a function of depth. These will serve as a benchmark against which the SeMSA will be compared. The 20–1000 Hz range is chosen as an ambitious target being both significantly broadband and low frequency.

### Average absorption against depth

**Figure 3:**
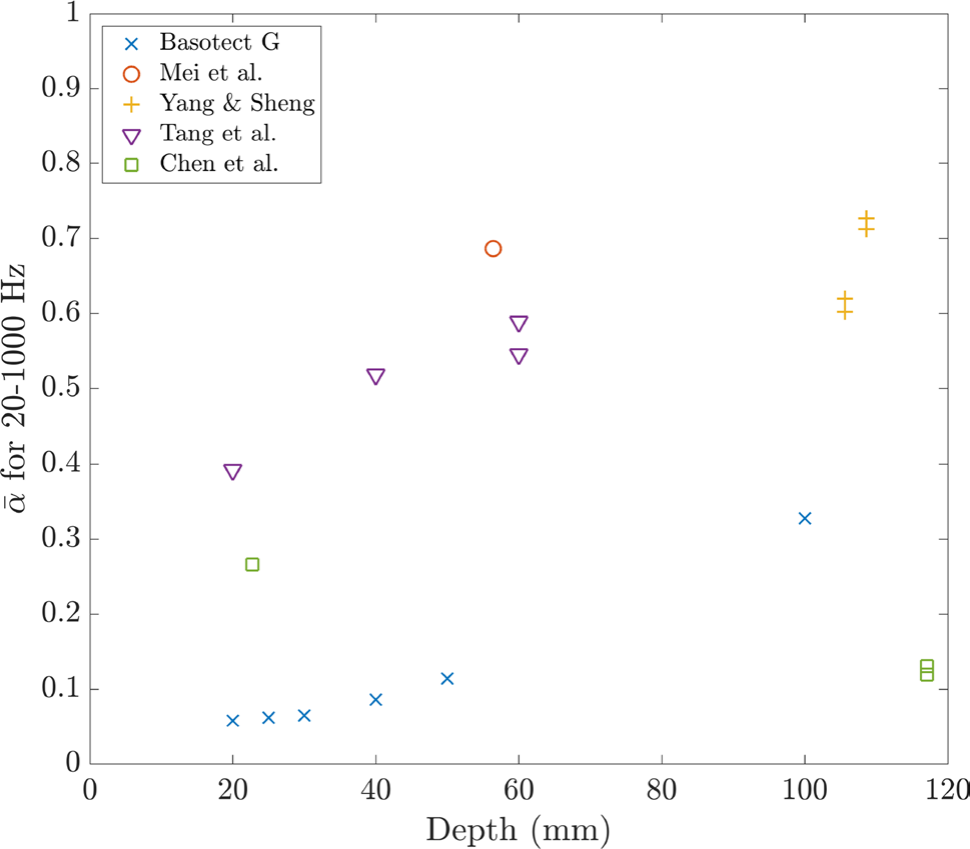
Literature survey. Average absorption in the range 20–1000 Hz as a function of absorber depth.

Figure 3 shows the average absorption against depth plotted for several absorbers in the range 20–1000 Hz. Data were extracted from figures in the literature using WebPlotDigitizer^13^. Where the data was not specified across the full range of 20–1000 Hz it was linearly extrapolated to $\alpha =0$ at 0 Hz. The plot contains multiple points for some designs at different depths because in some cases the papers presented multiple configurations of the design.

The DMR technology of Mei et al.^14^ achieved an average absorption of approximately 0.7 with an absorber of 56 mm deep although two layers of the metasurface were required. For shallower depths, the design of Tang et al.^15^, an MPP backed by intersecting honeycomb and corrugated structures that are also perforated performed relatively well. The yellow plus symbols are for a complex arrangement of 16 Fabry–Perot channels with a thin foam covering^12^ and these perform very well with high absorption values in this frequency range but data is available only for these greater depths. It would be useful to know how they would perform at shallower depths which would be necessary for many practical applications.

As expected, it can be seen that for this low-frequency range, acoustic foams such as Basotect G are not particularly effective whereas it is well understood that above 1 kHz and/or for greater thicknesses they perform better. The green squares show the results for the design of Chen et al.^11^ which consists of two axially coupled tubes coiled in a plane perpendicular to the incident wave.

## Equivalent circuit model of a SeMSA cell

**Figure 4:**
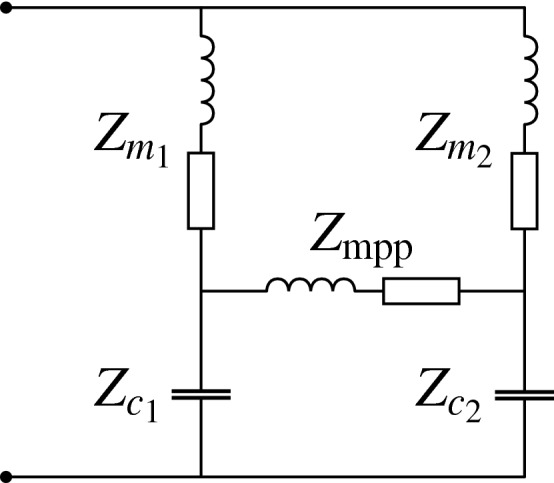
Equivalent circuit model of a SeMSA cell.

The two-segment SeMSA can be modelled using an equivalent circuit model similar to that described in Ref.^16^ where the authors present an absorber based on two MPPs with their backing chambers connected through a third MPP. The model presented in the current study is significantly different because it uses membranes on the surface rather than MPPs and it also does not require the two cavities to have the same dimensions. In fact, for the SeMSA, the different volumes are beneficial so that out-of-phase oscillation causes the necessary air pumping through the MPP holes. The equivalent circuit, shown in Fig. 4, uses the impedance analogy and the lumped element assumption is valid for small *ka* where *a* is a characteristic dimension. The resonant system formed by each membrane (m) and its chamber/cavity (c) is represented by the LRC circuit $Z_{m_i}+Z_{c_i}$ but there is the added complexity of the MPP coupling the two chambers, this is represented by $Z_\text {MPP}$. It should be noted that the impedances in the model are acoustic i.e. the ratio of complex pressure to volume velocity with units of kg/(m$^4$ s) rather than specific impedance^17,18^.

As discussed, rather than describing the full dynamics of the membrane, a simple limp-mass model is sufficient to capture the dominant first resonance of each membrane-cavity pair in the coupled system. The acoustic impedances of the limp-mass membranes are given by

1 $$\begin{aligned} Z_{m_i}=\xi _i+j\omega \rho _{m}\tau _{m_i}/A_i, \end{aligned}$$

where $\rho _{m}$ is the membrane density, $\tau _{m_i}$ is the thickness of membrane $i=1,2$ and $A_i$ is each membrane’s area. The $\xi _i$ term is a real, resistive term describing damping in the membrane.

The cavities are compliant as they are part of a Helmholtz resonator and together with the limp mass they form a resonant system. The stiffness of the cavities is given by $k_{c_i}=\rho _0 c_0^2/V_i$ where $V_i$ is the volume of each cavity. The acoustic impedance of the cavities is therefore given by:

2 $$\begin{aligned} Z_{c_i}=k_{c_i}/j\omega . \end{aligned}$$

Finally, the acoustic impedance of the MPP coupling the two cavities is considered. A solution for the propagation of sound in a narrow tube was first given by Rayleigh^19^ in 1877 and a simplified solution was subsequently provided by Crandall^20^ for tubes which are short compared to the wavelength. For MPPs, Crandall’s model can be used to calculate the MPP impedance and it is frequently used along with an end correction for pistonic sound radiation. More recent approximate models such as those proposed by Maa^1^ can also be used but with modern computing tools the calculation of the earlier more complex but complete analytical models is relatively simple. For consistency with the finite element model described later we will use the model implemented in COMSOL’s interior perforated plate (IPP) boundary condition for a thin plate, where (compared to viscous effects) thermal effects are negligible and where non-linear effects^21^ are ignored. For these conditions, the specific impedance of the MPP is given by:

3 $$\begin{aligned} \frac{z_\text {MPP}}{z_0}&=-\frac{jk}{\phi }\frac{J_0\left( \frac{d}{2}\sqrt{\frac{-j\omega }{\nu _0}}\right) }{J_2\left( \frac{d}{2}\sqrt{\frac{-j\omega }{\nu _0}}\right) }\left[ \tau +0.85d\cdot \Psi (\phi )\right] , \end{aligned}$$

where $j=\sqrt{-1}$, $z_0=\rho _0 c_0$ is the characteristic impedance of air with $\rho _0=1.225$ kg/m$^3$ and $c_0=343$ m/s, $k=\omega /c_0$ is the acoustic wavenumber, $\nu _0$ is the kinematic viscosity of air, *d* is the hole diameter, $\tau$ is the panel thickness, $J_n$ are the $n{\text {th}}$ order Bessel functions and $\phi$ is the porosity of the panel.

The Fok function, which accounts for hole-to-hole interaction^22^, is given by

4 $$\begin{aligned} \Psi (\phi )&=\sum _{F=0}^a a_F(\sqrt{\phi })^F, \end{aligned}$$

where the coefficients $a_F$ are given in Supplementary Table 1. Therefore, the acoustic impedance of the MPP coupling the two cavities is given by Eq. (3) divided by the area of the MPP: $A_\text {mpp}=L_c\cdot \min (D_1,D_2)$ where $L_c$ is the length of the chord and $D_{1,2}$ are the cavity depths.

To obtain the impedance across the two terminals of the equivalent circuit, the $\Delta -Y$ transform^23^ can be used on either of the two delta configurations in the circuit in Fig. 4^16^. Applying the transform to the bottom delta we obtain

5 $$\begin{aligned} Z_{A}&=Z_{c_1}Z_{c_2}/(Z_{c_1}+Z_{c_2}+Z_\text {mpp}) \end{aligned}$$

6 $$\begin{aligned} Z_{B}&=Z_{c_1}Z_\text {mpp}/(Z_{c_1}+Z_{c_2}+Z_\text {mpp}) \end{aligned}$$

7 $$\begin{aligned} Z_{C}&=Z_{c_2}Z_\text {mpp}/(Z_{c_1}+Z_{c_2}+Z_\text {mpp}). \end{aligned}$$

The remaining parallel branches can then be converted to series and the total specific impedance of the equivalent circuit is

8 $$\begin{aligned} z_\text {EC}&=\left( \frac{(Z_{m_1}+Z_B)(Z_{m_2}+Z_C)}{(Z_{m_1}+Z_{m_2}+Z_B+Z_c)}+Z_A\right) (A_1+A_2), \end{aligned}$$

where multiplying by the total projected area $A_1+A_2$ converts from acoustic impedance to specific acoustic impedance for use in the absorption calculation:

9 $$\begin{aligned} \alpha _\text {EC}=1-|R_\text {EC}|^2, \end{aligned}$$

where R is the reflection coefficient,

10 $$\begin{aligned} R_\text {EC}=(z_\text {EC}-z_0)/(z_\text {EC}+z_0). \end{aligned}$$

## Optimisation of a SeMSA cell

Given this expression for the specific acoustic impedance, Eq. (8), a cost function for optimisation can be defined which represents the average acoustic absorption in a chosen frequency range for a specific depth:

11 $$\begin{aligned} -\bar{\alpha }=-\frac{1}{\omega _2-\omega _1}\int _{\omega _1}^{\omega _2} 1-\left| \frac{z_\text {EC}-\rho _0 c_0}{z_\text {EC}+\rho _0 c_0}\right| ^2\, \text {d}\omega . \end{aligned}$$

The integrand calculates the absorption from the specific acoustic impedance, $z_\text {EC}$, and the negative appears at the beginning of the equation because although the algorithm seeks to minimise the cost function we wish to maximise the absorption coefficient. The bounds for the integral are $\omega _1=40\pi$ and $\omega _2=2000\pi$. A constrained optimisation tool such as Matlab’s *fmincon* can be used to minimise this cost function. Using this cost function the parameters of the SeMSA cell can be optimised.

**Figure 5:**
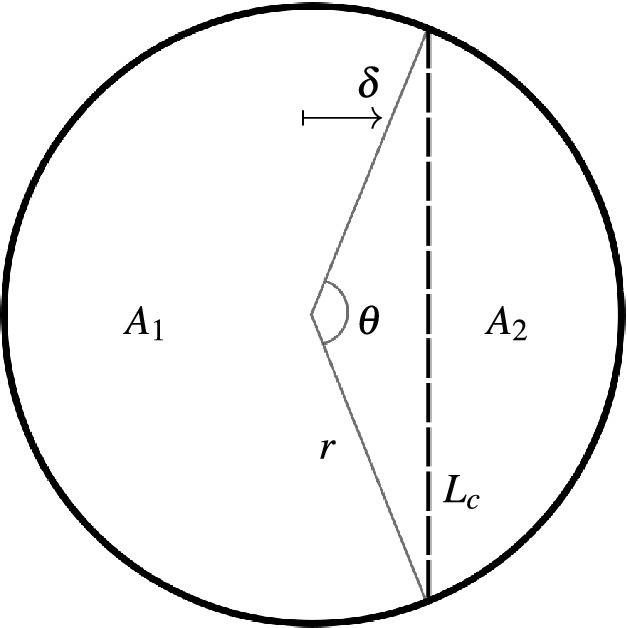
Cell geometry.

The response of the SeMSA cell is determined by a large number of parameters that can be included in the optimisation routine, however, there are dependencies between parameters that should be accounted for to improve the performance of the optimisation routine. The membrane areas $A_{1,2}$, MPP area $A_\text {MPP}$ and offset $\delta$ are linked through the angle $\theta$ defined between the ends of the chord $L_c$ made by the MPP and the origin as shown in Fig. 5.

The offset is given by

12 $$\begin{aligned} \delta&=r\cos (\theta /2). \end{aligned}$$

The area of the smaller membrane 2 is given by

13 $$\begin{aligned} A_2&=\frac{1}{2}r^2(\theta -\sin \theta ), \end{aligned}$$

from which we can calculate $A_1=\pi r^2-A_2$.

14 $$\begin{aligned} L_c&=2r\sin (\theta /2) \end{aligned}$$

is the length of the chord formed by the MPP and finally

15 $$\begin{aligned} A_\text {MPP}&=L_c\cdot \min (D_1,D_2) \end{aligned}$$

is the area of the MPP.

Regarding the two membrane damping values: $\xi _i$ given in Eq. (1), these are initially unknown as there is no such model for these. A preliminary test was performed on a single chamber SeMSA and the experimentally determined value for damping was used as the initial guess in the equivalent circuit (EC) model. Regarding the MPP, as we have seen in Eq. (3), the impedance of the MPP depends on the hole dimensions and the plate porosity which will depend on the plate’s area and so, the offset. The simplest way to link all these is to optimise the following parameters: $\theta$, $m_1$, $m_2$, $D_1$, $D_2$, $\tau$, *d* and the number of perforations, *n*. When the optimisation is performed, it is found that for a given maximum depth *D*, the depth of the two chambers will always tend to the maximum and so $D_{1,2}$ can be fixed as *D* to reduce the number of parameters. The perforation diameter tends towards its minimum allowable value.

**Figure 6:**
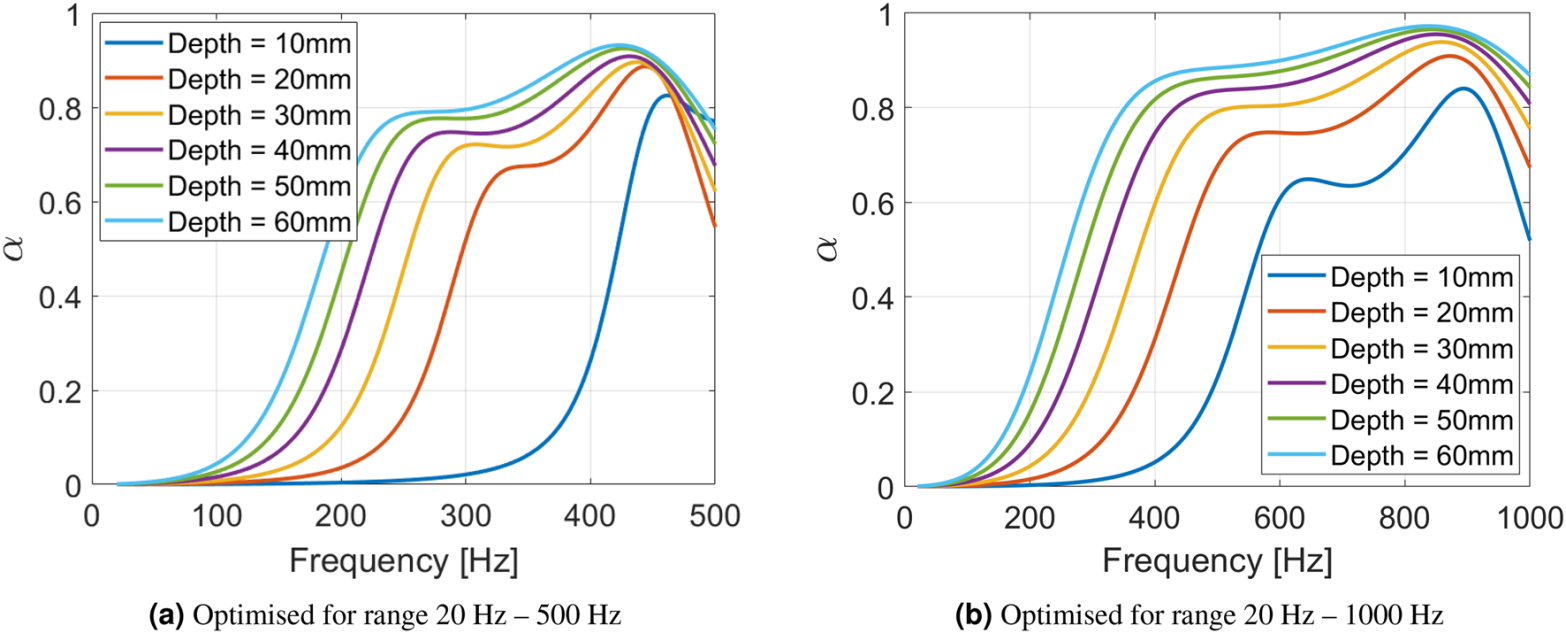
Absorption profiles of 40 mm diameter cylindrical SeMSA cells optimised for noise absorption for a range of cell depths.

Figure 6 shows the optimised SeMSA absorption curves for varying depths with Fig. 6a optimised for the range 20–500 Hz and Fig. 6b optimised for 20–1000 Hz. It can be seen that the optimal absorption curves have a similar profile. As the depth is increased the profile becomes broader and greater in amplitude, giving an improved average absorption. When the target range is reduced to 20–500 Hz the absorber still performs well, this is due to the flexibility afforded by tuning the system with added masses which can allow for very low-frequency resonances for small depths compared to classic Helmholtz resonator type designs for example. Typically in the acoustic metamaterial literature, efficient tonal technologies are termed “sub-wavelength” when the frequency of the tone of absorption has a wavelength significantly longer than a critical dimension (such as the depth) of the technology. The SeMSA being efficient over a broadband range of frequencies is obviously an improvement on tone-limited absorbers but in addition, can also be readily termed as “deep sub-wavelength”. Taking the 20 mm depth curve in Fig. 6a with an absorption coefficient of approximately 0.9 at 440 Hz, for example, we calculate it be 39 times shorter than the wavelength at which it is attenuating sound.

**Figure 7:**
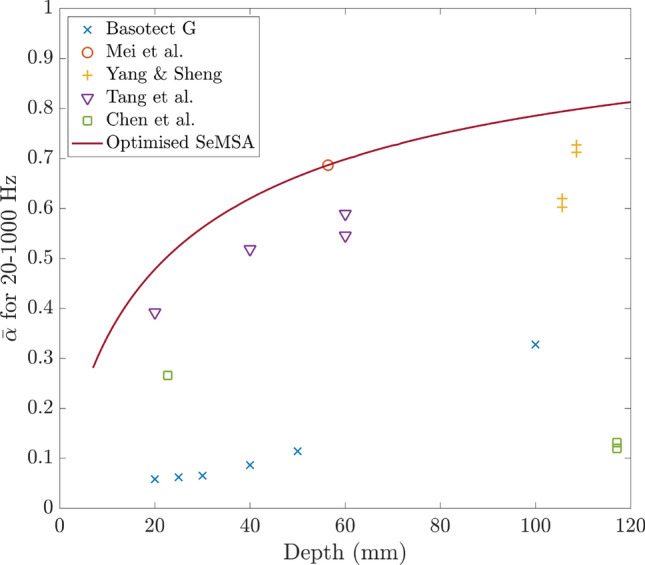
Average absorption in the range 20–1000 Hz against depth of the absorber with the solid line showing the optimised SeMSA absorber.

The solid red line in Fig. 7 shows the result of the optimisation for the SeMSA cell targeting 20–1000 Hz. Once again, for a specific depth, all parameters are allowed to vary in the optimisation process. This curve effectively plots the average absorption from the areas of the curves in Fig. 6b in addition to the other depths in the range up to 120 mm. From this curve, it can be seen that the optimised SeMSA cell outperforms all but one of the other broadband absorbers for the frequency range 20–1000 Hz for these depths demonstrating the significant performance of this novel technology.

## Finite element validation

The EC model enables design parameters of the SeMSA to be determined quickly and easily and lends itself well to the optimisation routine. Finite element analysis was also conducted using COMSOL Multiphysics to validate the EC model and also to provide additional insight into the physics of the technology. In the constructed FE model, the limp-mass behaviour of membranes, with or without platelet, was respected and no dissipation losses are accommodated at those locations, leaving the MPP as the sole means for absorption. In the FE model, to match the experimental tests performed, the SeMSA was located at the end of a hard-walled cylindrical tube of area A, with a 40 mm internal diameter. The SeMSA was excited by normally incident acoustic plane waves.

**Figure 8:**
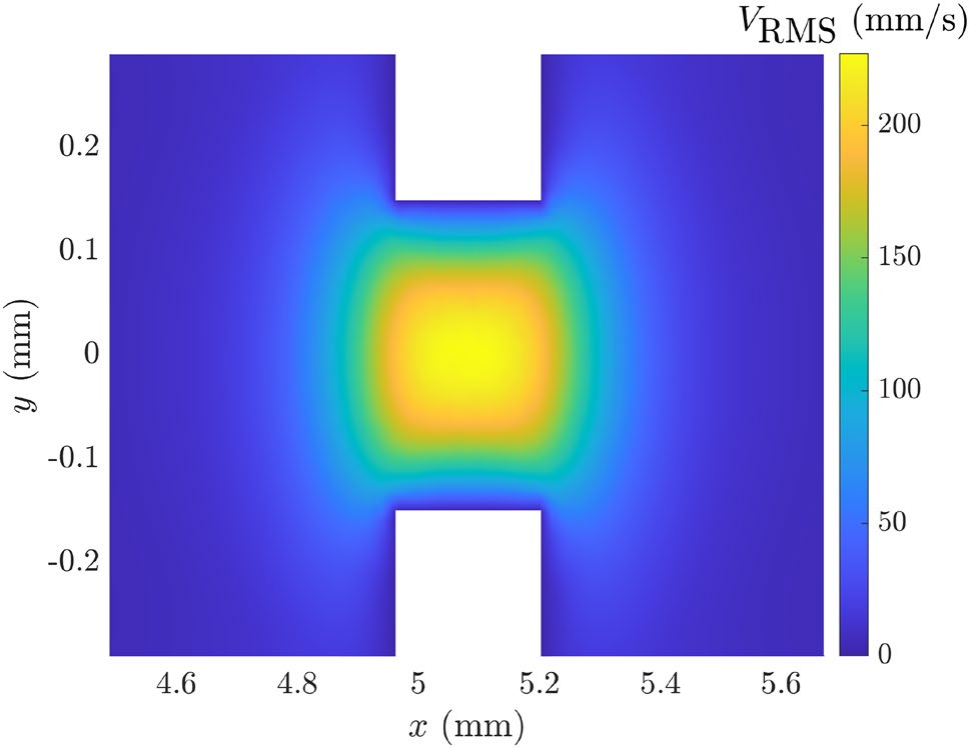
2D slice of $V_\text {RMS}$ in the region of a perforate.

The first FE model which was developed, referred to here as FE-TA, geometrically resolved all of the perforations in the MPP and used COMSOL’S thermo-viscous acoustics module. A close-up view of one of the perforations provides some insight into the absorption mechanism; Fig. 8 shows a 2D slice of the RMS velocity in the region of a perforation taken from the FE-TA SeMSA model. It can be seen that there is a large velocity gradient in the Stokes boundary layer formed within the perforate which is responsible for the large viscous losses seen in Figs. 6 and 7.

**Figure 9:**
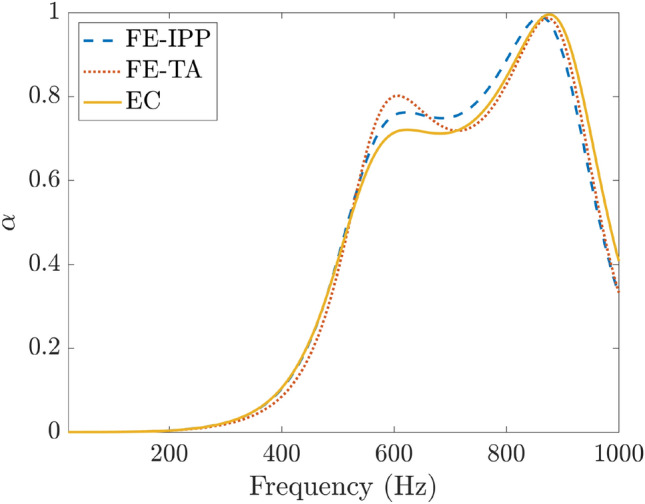
Comparison between the finite element interior perforated plate model (FE-IPP), finite element thermo-viscous acoustics model (FE-TA) and equivalent circuit model (EC). Optimised 15 mm parameters from Table 1.

A second, faster, FE model was also investigated which uses COMSOL’s interior perforated plate boundary condition, drawing on Eq. (3), to model the MPP, and is referred to here as FE-IPP. Both models are described in more detail in the Supplementary Information. Figure 9 compares the absorption coefficient of both the FE-TA and FE-IPP models to the EC model for a specific set of parameters as shown in Table 1—15 mm. To examine a particularly shallow depth, 15 mm was chosen for this particular SeMSA and it is examined also in the experimental section for comparison. The hole diameter of 0.3mm is chosen as it is considered to be the smallest reasonable diameter drill-bit which can be expected of a good quality workshop at the time of writing. Apart from their small characteristic differences, Fig. 9 shows that both FE models agree excellently with each other and with the EC model and through observation of this figure, in addition to other test points examined, it is reasonable to state that the EC model is valid at least for the range of depths of interest. To provide a measure of similarity for this example, the difference between the average absorption in the range 20–1000 Hz of the EC model and the FE-IPP model for this set of parameters is only 0.13%. Therefore, given that the interior perforated plate boundary condition (FE-IPP) is the far less computationally expensive method of the two, it can be used adequately when speed is necessary. The benefit of the FE models is that they can also be used for greater depth SeMSAs, if necessary, where the approximations associated with the lumped element assumption would be less valid. However, the authors are more interested in relatively shallow sound absorbers and thus in general either approach is feasible.

**Table 1:** SeMSA parameters: 15 mm and 25 mm deep cells.

Depth (mm)	$m_1$ (g)	$m_2$ (g)	$\tau$ (mm)	*d* (mm)	*n*	$\delta$ (mm)	$\phi$ (%)
15	0.28	0.56	0.15	0.3	4.3	5.1	0.05
25	0.2	0.54	0.1	0.3	6.9	6.05	0.1

**Figure 10:**
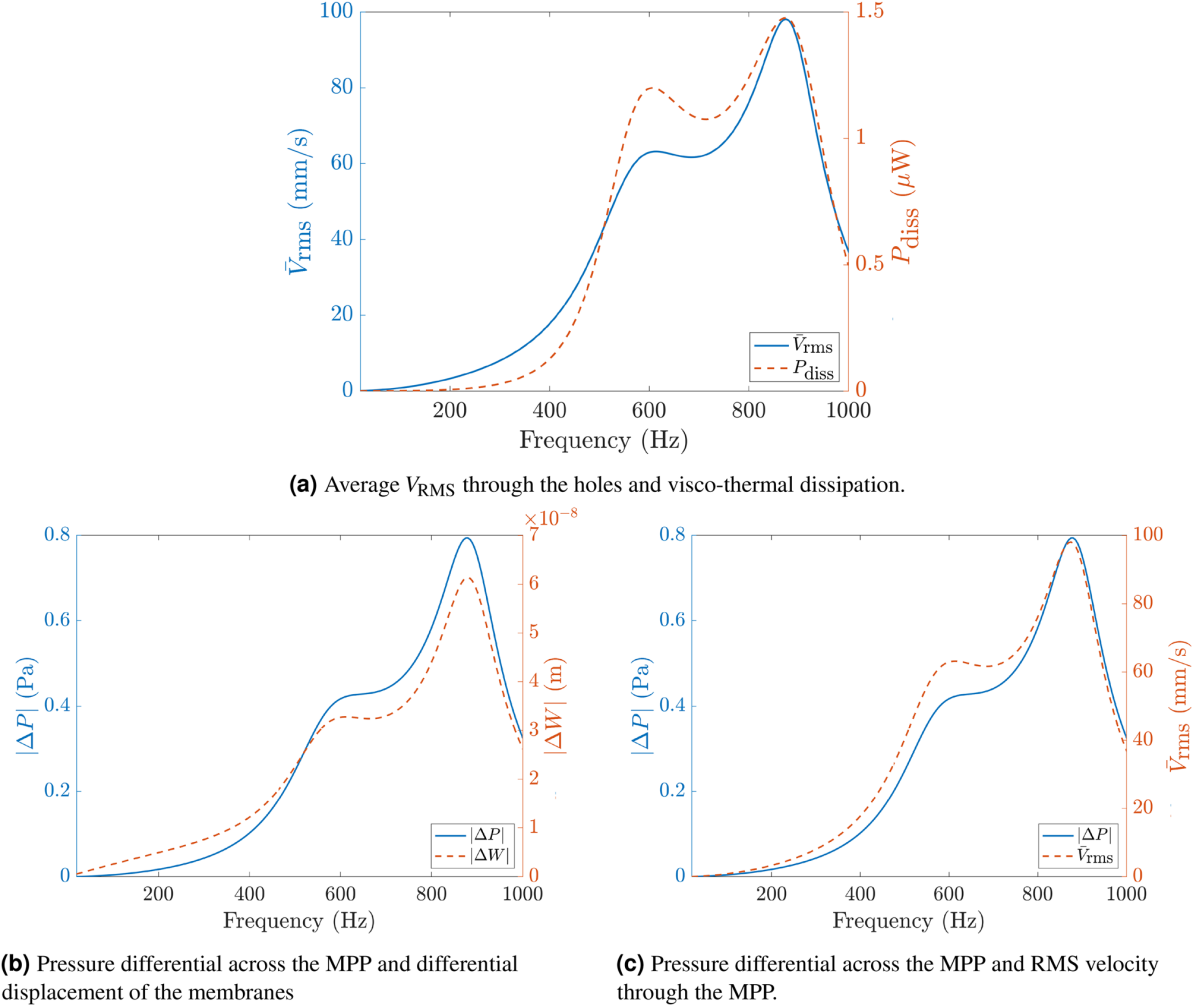
Finite element results.

Figure 10a shows the average $V_\text {RMS}$ in the perforates and the visco-thermal dissipation $P_\text {diss}$ in the hole which can be calculated from the definition of the absorption coefficient as

16 $$\begin{aligned} \alpha =\frac{P_\text {diss}}{P_\text {in}}=\frac{2z_0 P_\text {diss}}{p_0^2A}, \end{aligned}$$

where $P_\text {in}$ is the incident power, $p_0$ is the incident pressure and *A* is the inlet area. We see from the figure that the velocity and dissipation are closely related since a higher average $V_\text {RMS}$ produces a larger velocity gradient in the Stokes boundary layer and the only energy loss mechanism included in the FE model is through the microperforate.

Figure 10b,c show that the magnitude of the differential pressure across the MPP, the magnitude of the differential displacement of the two membranes and the average $V_\text {RMS}$ through the MPP are all closely related to each other and also to the corresponding absorption profile seen in Fig. 9. These plots support the physical premise of the SeMSA design: that the resonating membranes oscillating at either of two primary coupled frequencies, the frequencies of which can be lowered through the addition of platelets, create a pressure drop across the enclosed MPP. The pressure drop causes the enclosed air being pumped through the perforates from one chamber to the other resulting in a dissipative loss in the boundary layer of the holes when they are correctly sized.

## Experimental results

The SeMSA cells were tested in a normal-incidence impedance tube built to the ISO standard 10534-2:2001. The impedance tube has a circular cross-section of internal diameter 40 mm which gives a cut-on frequency of approximately 4 kHz. The noise signal is generated in Matlab and sent to a compression driver at the end of the tube via a DAQ and amplifier. The low-frequency limit for testing using this rig is 200 Hz. Below this, the results become noisy and unreliable due to the compression driver’s reduced low-frequency response.

**Figure 11:**
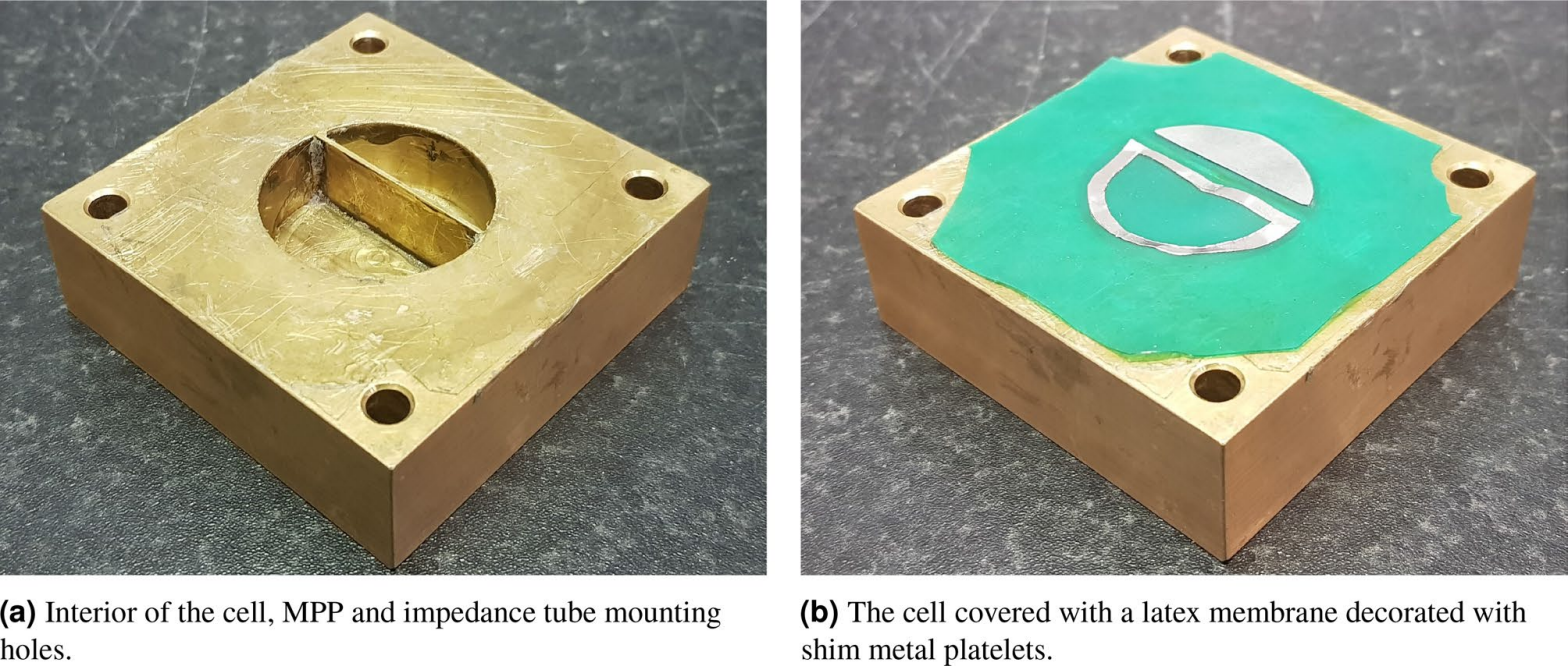
Prototype 15 mm SeMSA cell for experimental analysis.

Figure 11 shows a prototype SeMSA cell. The cell is circular to match the internal diameter of the impedance tube but it is machined from a block of brass to ensure no transmission through the SeMSA walls and so that it may easily be secured to the end of the tube. The decorated membrane is made from a sheet of latex rubber $\tau _m=0.18$ mm thick carefully glued down to the edges of the SeMSA cell including along the top of the MPP. To tailor the response of the absorber, the mass of each segment’s membrane can be adjusted by glueing on material such as thin metal platelets. In our case, we used thin “shim” metal and cut it to shape. As already mentioned, the added mass must be sized slightly smaller than the cell segment so that there is a latex ‘hinge’ to help ensure limp mass behaviour. The masses were carefully cut and measured with precise electronic scales to help ensure accuracy. For very light masses, material was removed from the centre of the shim metal. This allowed the mass to be reduced whilst ensuring material was located close to the perimeter, as can be seen in Fig. 11b. The mass of the glue was included in the calculations. The MPP was carefully cut to length for the correct offset and then glued in place, care taken to ensure that excess glue did not reduce chamber volumes or block perforations.

An initial measurement was performed on a 25 mm deep SeMSA cell with the optimised parameters shown in Table 1—25 mm. A plate thickness of $\tau =0.1$ mm is extremely thin and would not have suitable rigidity to machine especially when drilling the 0.3 mm perforations. To address this, the perforations were drilled in two stages into a 1 mm thick plate which was used in all such experiments. An initial counter-bore using a larger diameter drill bit was used to reduce the thickness of the plate in the area where the perforation was to be. A second hole was then drilled with the 0.3 mm diameter drill bit. A high precision optical microscope was used to inspect these holes and measurements showed the process to be successful, however, from the microscope images the average micro-perforate hole diameter was measured to be 0.27 mm rather than 0.3 mm.

**Figure 12:**
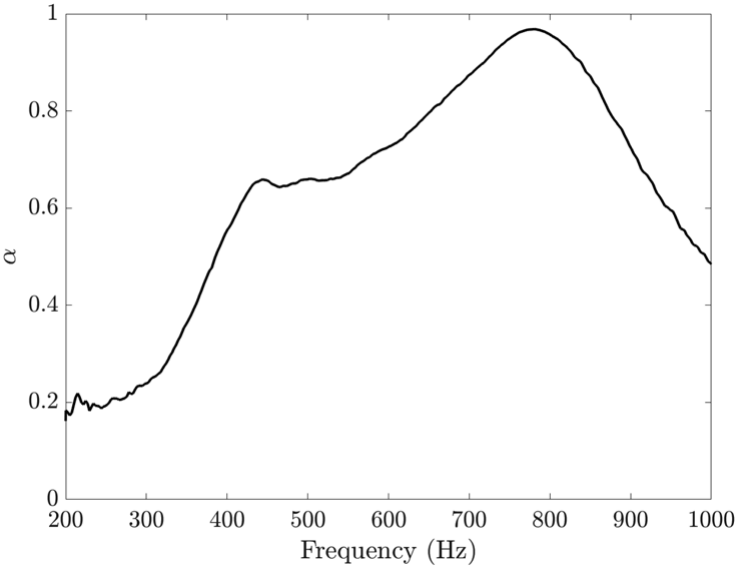
Experimental results for a 25 mm deep SeMSA cell. $\bar{\alpha }=0.53$.

Figure 12 shows the absorption curve which appears to consist of two merged peaks resulting in broad, high and even near-perfect absorption at 740 Hz. The average absorption in the range 20–1000 Hz was calculated to be 0.53. where the absorption values were linearly extrapolated from the value at 200 Hz to $\alpha =0$ at 0 Hz.

**Figure 13:**
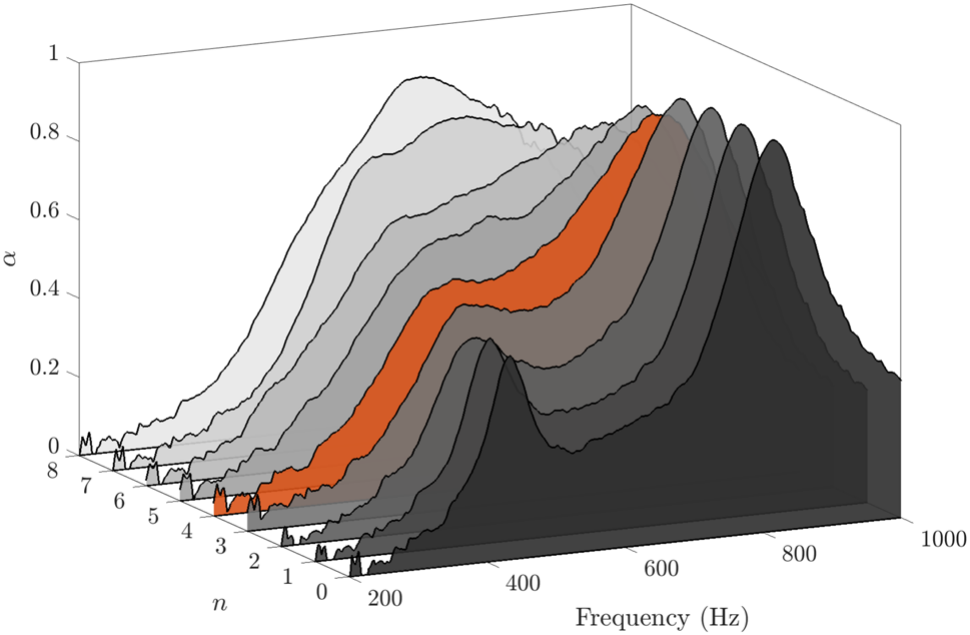
Experimental absorption profiles of 15 mm SeMSA cell. MPP porosity is varied by changing number of holes. $\bar{\alpha }=0.4$ for 4 hole case.

A second SeMSA was also tested to gain some sense of performance for a very shallow technology. The second depth was only 15 mm but was again optimised over the wide range of 20–1000 Hz, see Table 1. The red curve in Fig. 13 presents the absorption coefficient results of this SeMSA. To gain some additional information concerning the sensitivity of the SeMSA to the porosity, four extra holes of the optimised dimensions were drilled in addition to the four (actually 4.3) calculated from the optimisation process. This SeMSA was then tested repeatedly with the number of holes being reduced in each test by simply temporarily blocking them. We verify that the optimised number of holes produces the maximum absorption value: $\bar{\alpha }=0.4$, with the 5 and 6 holes cases resulting in very similar values. The exercise also allows us to get a sense of how the SeMSA behaves as it transitions from two independent chambers/resonators when all the holes are blocked, $n=0$, towards increasingly coupled chambers. With increasing hole number, we observe the two main peaks associated with the two independent resonators approach each other in frequency and magnitude, resulting in a broad high-level absorption band range.

**Figure 14:**
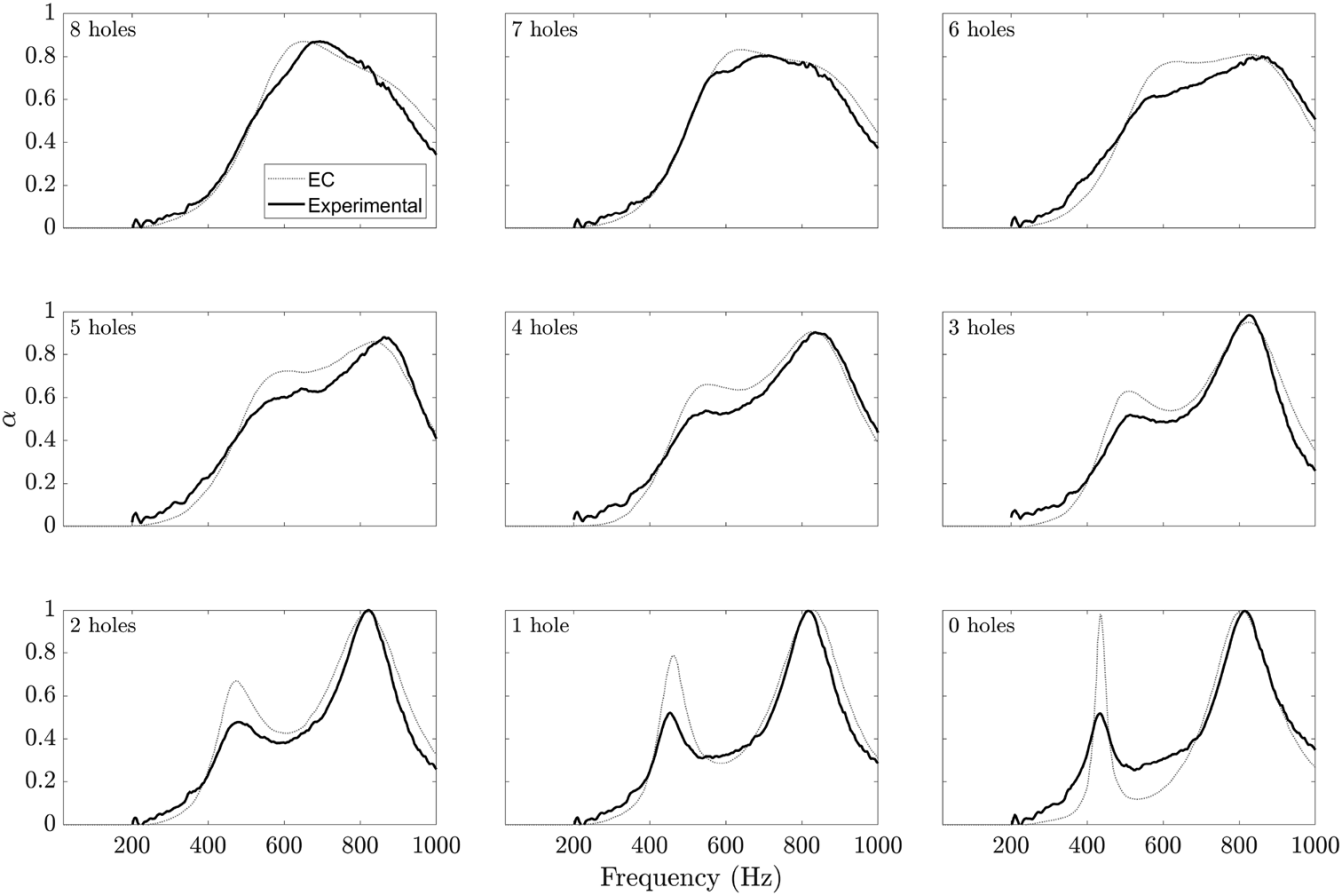
Absorption profiles of 15 mm SeMSA cell. EC model vs experimental results.

To further explore this behaviour the same exercise was conducted with the EC model, (grey dotted line) using the optimal parameters from Table 1, whose curves are superimposed over the experimental results in Fig. 14. Several interesting observations can be made. For zero holes, the EC model displays two peaks of equal magnitude and perfect absorption in the frequency range of interest. The lower frequency peak corresponds to the smaller chamber over which the greater mass is fixed. This frequency corresponds very well with the lower frequency experimental peak although with greater magnitude. This peak quickly becomes less distinct as the number of holes increases. Also, as the number of holes increases, the low-frequency EC model peak gradually increases in frequency and continuously broadens in frequency range. Its amplitude initially decreases before increasing again. The behaviour of the higher frequency EC model peak is somewhat different. Its amplitude remains at perfect absorption until there are four holes and its frequency remains steady. However, as the two peaks merge its amplitude drops leading to a broad combined hump. The experimental results are an excellent match in particular for the high-frequency peak. For small hole numbers, the experiment also matches the low-frequency value well but does not reproduce the high amplitude. Of the two EC model peaks, this low frequency peak is much narrower and thus most likely has lower damping. The damping in the experiment is suspected to be higher and therefore does not achieve the same magnitude.

Comparing the EC model to the experimental result for $n=4$, we see the two sets of curves have very similar form but the experimental plot is lower in magnitude at the lower frequency. This discrepancy may be due to inaccuracies in the mass of the membranes, platelets or adhesive, and deviation from ideal limp mass behaviour in the regions around the membrane edges. Also, the depth of the cavity may not precisely match the target depth. As the membrane is deliberately not tensioned to ensure limp mass behaviour, this may cause the membrane to sag under its own weight in quiescent conditions thus reducing the cavity’s volume. In addition, an integer number of holes: 4 was chosen as opposed to 4.3 as provided by the optimisation routine and the diameter of those was 0.27 mm as opposed to 0.3 mm. Finally, it is uncertain if a precise hole depth equal to 0.15 mm was attained.

Another explanation for the discrepancy between the four-hole experimental and EC model results is that the values for damping used in the model may differ from the actual values for the two membranes in the experiment. The EC model curve certainly models a particular SeMSA for the damping values used and the curve is valid, but it does not necessarily model the performance of the particular SeMSA used in the experiment. It is difficult to know a priori what these values would be without an accurate damping model, which was not pursued in this case, instead the values were determined from the experimental result itself.

**Figure 15:**
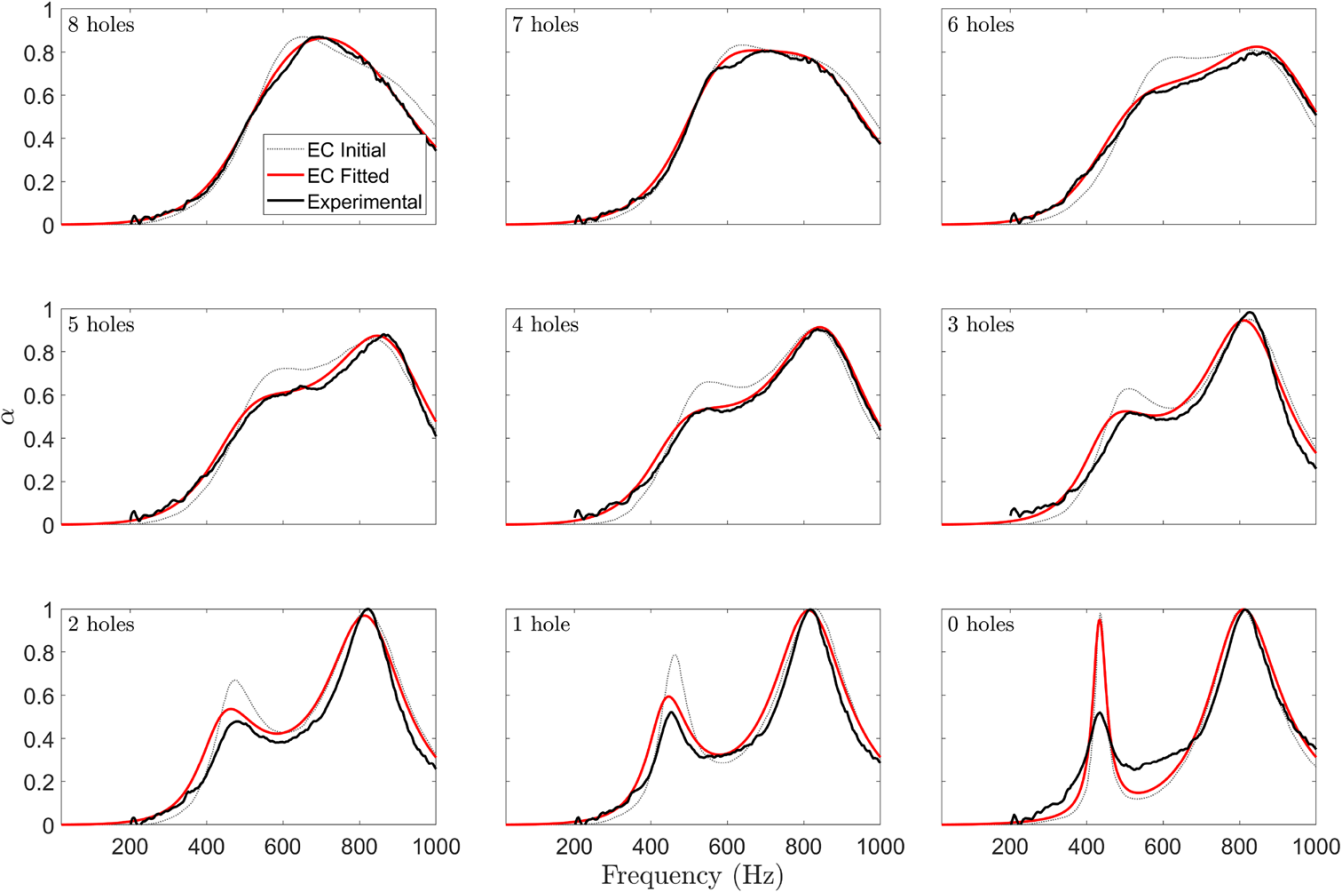
Absorption profiles of 15 mm SeMSA cell. EC model vs experimental results vs Global Fit for damping.

**Figure 16:**
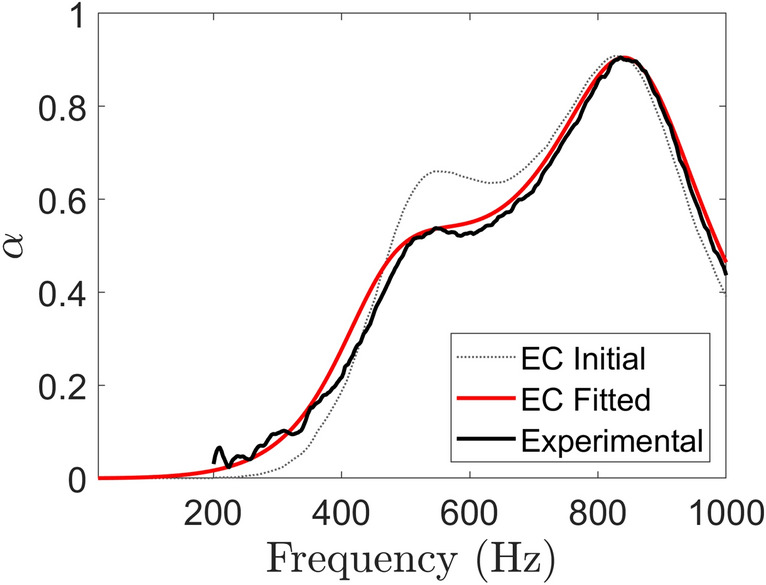
Absorption profiles of 4 hole, 15 mm SeMSA cell. Damping value fit of EC model to the experimental result.

Using the EC model curves in Fig. 14 as a starting point, the model was fit to the experimental result by minimising the sum of the squared error between the experimental absorption and the equivalent circuit’s absorption. In Fig. 14 it is important to remember that the hole number is the only experimental parameter which changes from one plot to the next, therefore, rather than fitting each EC model curve individually to each experimental curve, the results were fitted as a whole i.e. the optimisation was performed on the cost function given by the error produced by summing the error of all the results. During the fitting process, the two membrane damping terms were left free to vary independently. Figure 15, shows the fit using the new damping terms. Therefore, when looking at the fitted EC model plots (red curves) in Fig. 15, as for the experimental plots, it is only the number of holes that changes from one plot to the next. By fitting the model to all nine curves demonstrates that the model generalises well. This process also ensures that we have rigorously avoided “overfitting” which might occur by simply trying to fit the EC model to the experimental result for only the four hole case. In order to improve clarity, the four hole plot corresponding to our experiment is extracted from the grid of plots in Fig. 15 and presented in isolation in Fig. 16. It can be seen that an excellent fit is achieved and thus good estimates of the true damping values are semi-empirically calculated.

**Figure 17:**
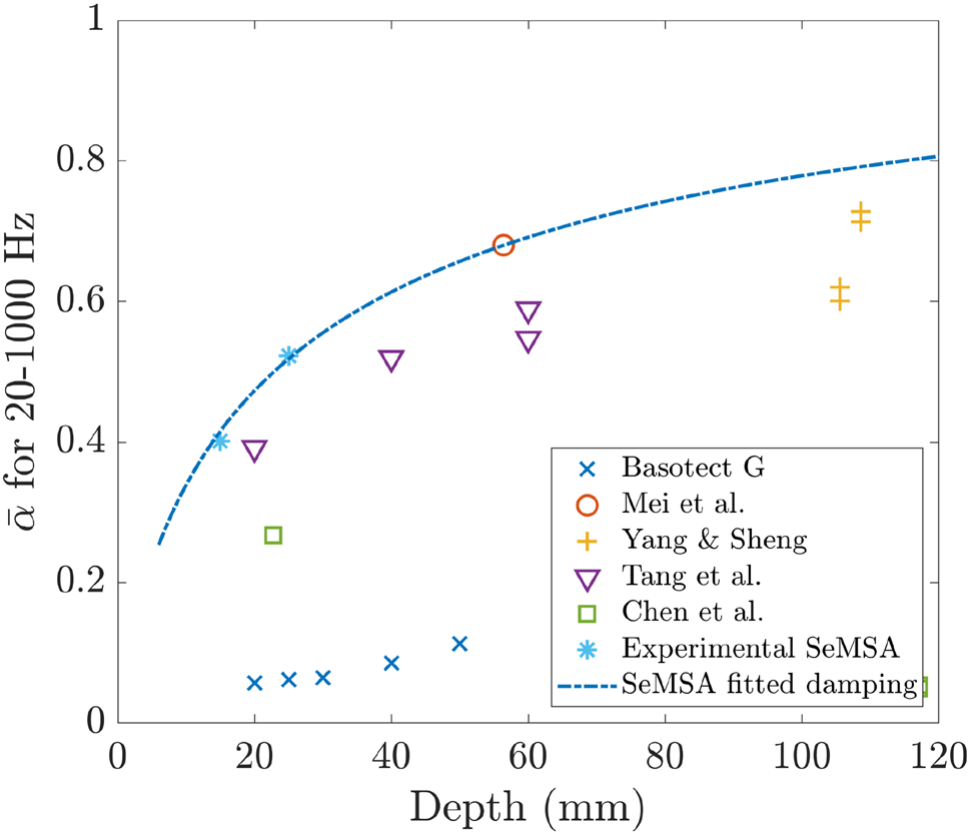
Average absorption in the range 20–1000 Hz against depth of the absorber. Solid line shows optimised SeMSA EC model with corrected damping values. Blue asterisks show experimental results at 15 mm and 25 mm.

Using these corrected values for membrane damping a new optimised EC model SeMSA curve is plotted as a function of depth and compared to the previously mentioned examples from the literature in Fig. 17. Both values for the experimental results, at 15 mm and 25 mm, are also plotted and they now agree extremely closely with the model. This plot demonstrates that the SeMSA can be modelled successfully and that it is the best low-frequency broadband sound absorber compared to the literature (to the authors best knowledge) in the frequency band 20–1000 Hz. The exception to this is the 56 mm Mei et al.^7^ result where the SeMSA is predicted to have the same absorption.

**Figure 18:**
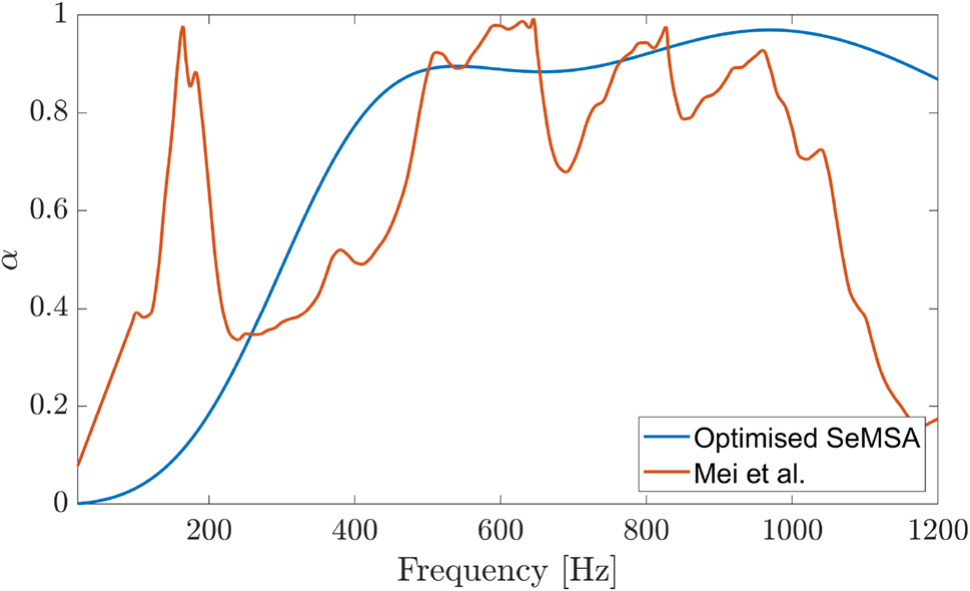
SeMSA V Mei 56 mm. SeMSA: $\bar{\alpha }=0.71$, Mei et al.: $\bar{\alpha }=0.64.$

A further step was taken in order to compare the SeMSA with the Mei et al. super absorber. As the authors actually provide results for their technology up to 1.2 kHz: Mei et al.— Fig. 4b^7^, Fig. 18 shows a direct comparison between the SeMSA absorber, optimised this time for the 20 Hz—1.2 kHz range, with the experimental result of the Mei et al. absorber. Given that the absorption coefficient of the SeMSA remains high in the 1–1.2 kHz range, its average absorption over the extended range from 20 Hz to 1.2 kHz is actually significantly higher at $\bar{\alpha }=0.71$, compared to the Mei et al. technology with an average absorption of $\bar{\alpha }=0.64$.

## Conclusions

A novel sound absorber called the SeMSA is introduced which is extremely effective at low-frequency broadband sound absorption and which can achieve this at deep sub-wavelength thicknesses. The technology is compared to other absorbers to be found in the literature and the SeMSA outperforms all but one in the 20 Hz–1000 Hz range for depths of up to 120 mm. When the upper frequency range was increased to 1.2 kHz, to match the range of Mei et al., it was found that the SeMSA outperformed this technology also. These comparisons were verified through analytical, finite element and experimental analyses. The absorber is constructed of two parallel air cavities which are coupled by a micro-perforated plate. The cavities are sealed with mass decorated limp membranes. The new technology, therefore, combines the highly efficient visco-thermal loss mechanism of an MPP and successfully lowers its frequency response, for this depth, by combining it with decorated mass resonators. Given that the MPP is sealed behind the membrane, it is therefore not subject to blockage and hence performance deterioration contrasting with other MPP designs. The absorber merges two principle broad absorption peaks whose bandwidths, peak levels and centre frequencies are controlled by the physical properties of the cell such as the membrane masses, plate porosity, plate offset and cavity depths. This performance can be modelled analytically using an equivalent circuit model, and this model can be used to design an optimal cell for absorption in a prescribed frequency range. The optimisation routine can take into account physical constraints for a real application such as manufacturing limitations or available volume. Whereas the dissipative properties of the MPP can be modelled, the material damping in the limp membrane is more difficult to predict. A semi-empirical procedure is developed which successfully estimates the damping coefficients and hence the optimisation routine can take the case-specific damping into account.

This paper has focused on a cylindrical SeMSA geometry, but the equivalent circuit model makes no assumption of cell geometry and so may be applied to other cell geometries such as those that tessellate, e.g. rectangular or hexagonal.

The authors believe that acoustic metamaterial research would benefit from benchmarking challenges, as are to be found in other domains^24,25^, so that advancements can be compared to one another. Average absorption in a frequency range, e.g. 20 Hz–1 kHz is an example used here but of course others are possible.

## Supplementary information

Supplementary Information.
