# Supplementary material for: SeMSA: a compact super absorber optimised for broadband, low-frequency noise attenuation
Source: Sci Rep. 2020 Oct 21;10:17967. doi: 10.1038/s41598-020-73933-0 (PMC7578840; doi:10.1038/s41598-020-73933-0)
Supplement: Supplementary file 1 — Supplementary Information. [file 41598_2020_73933_MOESM1_ESM.pdf]

SeMSA: a compact super absorber optimised for broadband, low-frequency noise attenuation.

Andrew McKay[1,*], **Ian Davis**[2], **Jack Killeen**[1], **and Gareth J. Bennett**[1,*]

[1]Department of Mechanical & Manufacturing Engineering, Trinity College Dublin, the University of Dublin, Ireland
[2]Efficient Energy Transfer (ηET) Department, Nokia Bell Labs, Blanchardstown Business & Technology Park, Dublin 15, Ireland
[*]admckay2@gmail.com, gareth.bennett@tcd.ie

June 26, 2020

A Fok function coefficients

a_0	a_1	a_2	a_3	a_4	a_5	a_6	a_7	a_8
1	-1.4092	0	0.33818	0	0.06793	-0.02287	0.063015	-0.01614

Table 1. Coefficients of the Fok function

B Finite element model

The finite element model used to verify the equivalent circuit model and to provide insight into the physics of the system was performed using COMSOL Multiphysics with the acoustics and structural dynamics packages. Figure 1 shows the geometry of the COMSOL model.

The geometry is cylindrical with a diameter of 40 mm to match the experimental geometry. The domain below the blue highlighted boundary is pressure acoustics and its bottom face is set to plane wave radiation to absorb reflected sound and it has an incident pressure field subnode to provide the incident pressure.

The blue highlighted boundary is the membrane, defined using the structural dynamics membrane interface. The different membrane masses are produced by using the 'change thickness' option, this could also be achieved by having different densities. Little variation is seen between the results when the membrane is fixed or free around the edges but they are expected to diverge for higher pressure amplitudes.

Above the membrane are the two chambers which are in the pressure acoustics domain. For the FE-TA model, these chambers are separated by a slice removed from the domain of the same thickness as the MPP, small cylindrical connections across this slice are the microperforates. The holes of the microperforated panel are in the thermo-viscous acoustics domain. The thermo-viscous acoustics region of each hole is a 'dumbbell' shape where the thinner middle section has the same dimensions as the hole and the two wider end sections intrude into the pressure acoustics region to capture any viscous end effects. The shape can be seen in the 2D velocity slice shown in figure 8. Alternatively, rather than simulating the individual holes, the interior perforated plate boundary condition can be used by simply defining a boundary to separate the chambers and applying the boundary condition. This FE-IPP model is significantly quicker to compute and gives comparable results to the FE-TA model, see figure 9.

The absorption calculation is performed by calculating the impedance at the inlet on the bottom surface and using the impedance translation theorem to calculate the surface impedance of the SeMSA cell. This surface impedance is then used in the calculation of the reflection and absorption coefficients. Alternatively, the acoustics domain outside of the SeMSA cell can be removed and pressure can be applied directly to the membrane

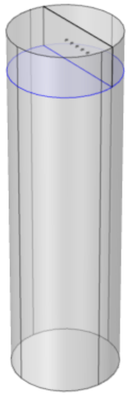

Figure 1. COMSOL geometry

B.1 Calculations from finite element model

In figure 10a, the RMS velocity is calculated across the interior perforated plate boundary condition. The RMS velocity is given by

$$V_{\text{RMS}} = \frac{1}{\phi\sqrt{2}}\left|\frac{P_+ - P_-}{Z_{\text{MPP}}}\right| \tag{1}$$

where P_+ and P_- are the complex pressure values either side of the boundary. In COMSOL these pressures either side of the boundary are given by up(acpr.p_t) and down(acpr.p_t). When the interior perforated boundary condition is used in COMSOL, the MPP impedance is available as the variable acpr.Zi. The result is divided by $\sqrt{2}$ to convert to RMS and ϕ to convert from velocity across the whole interior perforated plate boundary condition to the average velocity in the holes of a plate with porosity ϕ.

In figure 10a, the visco-thermal dissipation is calculated from a SeMSA cell modelled with individual perforations rather than the interior perforated plate boundary condition. The dissipation value is calculated by defining an integration operation in the thermo-viscous acoustics region of all the holes. The visco-thermal dissipation is then given by intop1(ta.diss_tot).

In figure 10b the values $|\Delta P|$ and $|\Delta W|$ are obtained in a similar way to V_{RMS}. Both are calculated from the model which uses the interior perforated panel boundary condition for the MPP.

$$|\Delta P| = |P_+ - P_-| \tag{2}$$

and the differential displacement of the two membranes is calculated from the average membrane Z displacement W of the two membranes:

$$|\Delta W| = |W_1 - W_2|\,. \tag{3}$$

For limp mass behaviour, the displacement of the membrane can be represented by the motion of a point on the membrane but in COMSOL it is simpler to use an average operator for each membrane so that W_1=aveop1(w) and W_2=aveop2(w).
